# Mechanism of Sporicidal Activity for the Synergistic Combination of Peracetic Acid and Hydrogen Peroxide

**DOI:** 10.1128/AEM.03010-15

**Published:** 2016-02-05

**Authors:** Mark J. Leggett, J. Spencer Schwarz, Peter A. Burke, Gerald McDonnell, Stephen P. Denyer, Jean-Yves Maillard

**Affiliations:** a Cardiff School of Pharmacy and Pharmaceutical Sciences, Cardiff University, Cardiff, United Kingdom; b Steris Corporation, St. Louis, Missouri, USA; c Steris Corporation, Mentor, Ohio, USA; University of Minnesota

## Abstract

There is still great interest in controlling bacterial endospores. The use of chemical disinfectants and, notably, oxidizing agents to sterilize medical devices is increasing. With this in mind, hydrogen peroxide (H_2_O_2_) and peracetic acid (PAA) have been used in combination, but until now there has been no explanation for the observed increase in sporicidal activity. This study provides information on the mechanism of synergistic interaction of PAA and H_2_O_2_ against bacterial spores. We performed investigations of the efficacies of different combinations, including pretreatments with the two oxidizers, against wild-type spores and a range of spore mutants deficient in the spore coat or small acid-soluble spore proteins. The concentrations of the two biocides were also measured in the reaction vessels, enabling the assessment of any shift from H_2_O_2_ to PAA formation. This study confirmed the synergistic activity of the combination of H_2_O_2_ and PAA. However, we observed that the sporicidal activity of the combination is largely due to PAA and not H_2_O_2_. Furthermore, we observed that the synergistic combination was based on H_2_O_2_ compromising the spore coat, which was the main spore resistance factor, likely allowing better penetration of PAA and resulting in the increased sporicidal activity.

## INTRODUCTION

Bacterial spores are a highly resistant cell type formed when certain members of the Firmicutes, e.g., Bacillus and Clostridium spp., encounter environmental stress, commonly nutrient starvation. The bacterial spore structure differs markedly from the structure of the vegetative cell, and the differences confer upon the spore remarkable resistance to many environmental stresses, including extremes of temperature, radiation, and chemical assault (1, 2). These heightened resistance properties also confer upon the spore significant resistance to disinfection and sterilization procedures relative to that of their vegetative cell counterparts.

Relatively few chemical antimicrobials have genuine sporicidal activity, and those which do are principally among the alkylating and oxidizing agents (3, 4). There has been considerable interest in the mechanisms of activity of and spore resistance to such sporicidal agents, in the hope that a better understanding of such treatments will allow for improved disinfection procedures in the future (2).

Oxidizing agents are widely used for the control of spore-forming organisms, with chlorine-based disinfectants finding particularly widespread use (3). Two other oxidizing agents, peracetic acid (PAA) and hydrogen peroxide (H_2_O_2_), are also sporicidal (5–7) and are used for industrial process decontamination, food decontamination, and medical device disinfection/sterilization and, increasingly, as environmental disinfectants.

It is well cited that PAA and H_2_O_2_ in combination (P/H) act synergistically to dramatically improve their bactericidal and sporicidal activities relative to those of either agent used alone (8–10). Indeed, several products exploiting this combination are currently approved by the U.S. Food and Drug Administration for use as sterilants and/or high-level disinfectants (11). Despite the relatively common use of this combination and the well-studied mechanisms of activity of these and other oxidizing agents (1, 2), little is known about the mechanism of the interaction of these two biocides. Indeed, it is usually remarked that synergy may occur between combinations of antimicrobials which affect different sites within the target organism (12). As such, it is perhaps surprising that PAA and H_2_O_2_ act synergistically, given that both have been proposed to kill spores, at least in part, by damaging the inner membrane (7, 13, 14).

This study details information regarding the potential mechanism of the synergistic interaction of PAA and H_2_O_2_ applied against bacterial spores.

## MATERIALS AND METHODS

### Bacterial strains, growth conditions, and spore preparation.

The Bacillus subtilis strains used in this study (kindly provided by P. Setlow, UConn Health, Farmington, CT) are isogenic derivatives of strain 168. Strain PS533 (wild type [WT]) contains plasmid pUB110, which carries a kanamycin (Km) resistance marker. Strain PS578 (α^−^ β^−^) also contains pUB110, but it lacks the genes that encode the two major α/β-type small acid-soluble spore proteins (SASPs), i.e., *sspA* and *sspB* (15). Strain PS3394 (CotE^−^) is defective in the *cotE* gene, which has mostly been replaced by a tetracycline (Tc) resistance cassette, resulting in a defective spore coat. PS3394 also contains plasmid pUB110 (16).

All strains were routinely grown in LB medium (agar or broth; Fisher, United Kingdom), with or without antibiotic supplements (for PS533 and PS578, Km at 10 μg/ml; and for PS3394, Km at 10 μg/ml and Tc at 5 μg/ml). Spores were prepared on 2× SG medium agar (17) without antibiotic selection by inoculating plates with 0.2 ml of a growing culture of the relevant strain (at an optical density at 600 nm [OD_600_] of ∼1) and incubating them at 37°C for 3 to 5 days. All growth was scraped from the plates and cleaned as described previously (18).

### Microbicide testing.

Spore survival was routinely assessed at room temperature (∼25°C) and from a starting spore titer of 10^9^ CFU/ml. Microbicides used were PAA in water (Sigma-Aldrich, United Kingdom) and H_2_O_2_ in water (Steris Corp.), at the concentrations indicated in the text. After relevant contact times, samples were removed and diluted 1:10 in neutralizing solution (20 g/liter sodium thiosulfate with 500 U/ml catalase) at room temperature for a minimum of 10 min. The neutralization method was validated against the concentrations of PAA and H_2_O_2_ used for the assay (results not shown). Neutralized aliquots were serially diluted (1:10) in sterile deionized water, plated onto LB agar (without antibiotics), and incubated at 37°C for 16 to 30 h before counting survivors. For experiments with spore populations that had been partially inactivated (∼90% kill), test solutions were neutralized as described above and then filtered to harvest spores. Samples were then treated two more times with the same neutralizer to ensure thorough inactivation of the microbicides (as described in reference 13) before washing three times in sterile phosphate-buffered saline and resuspension in sterile deionized water.

Spore kill data were plotted as survival curves and used to calculate the concentration exponent (η) of each microbicide. Due to the very low activities of the individual microbicides at lower concentrations, log_10_ reduction values for PAA concentrations of <0.08% (wt/vol) were extrapolated based on the η values obtained for PAA concentrations (wt/vol) of 0.08% (pH 3.7), 0.16% (pH 3.0), and 0.32% (pH 2.7). Similarly, for H_2_O_2_, log_10_ reduction values for concentrations of <0.8% (wt/vol) were extrapolated based on the η values obtained for H_2_O_2_ concentrations (wt/vol) of 0.8% (pH 4.5), 1.6% (pH 4.0), 3.2% (pH 3.8), and 6.4% (pH 3.2).

### Assay of PAA and H_2_O_2_ during microbicide treatment of spores.

For some experiments, the PAA and H_2_O_2_ contents within mixtures of PAA, H_2_O_2_, and bacterial spores were determined 0, 5, and 15 min after mixing by using the method of Bruce Ritts (personal communication), as described below. Reactions were conducted at room temperature (22 to 24°C), and reaction mixtures contained ∼10^8^ PS533 (WT) spores and combinations of H_2_O_2_ and PAA at either 1%:0.05% (pH 3.1), 3%:0.05% (pH 3.0), or 1%:0.15% (pH 2.8). PAA and H_2_O_2_ determinations were completed in parallel by using portions of the same aliquot at each time point. To assay for PAA, an aliquot of the reaction mixture was added to a solution of *N*,*N*-diethyl-*p*-phenylene-diamine (DPD; 15 mM DPD and 1.7 mM EDTA) and halogen phosphate buffer (HPB; 211 mM Na_2_HPO_4_, 72 mM NaH_2_PO_4_·H_2_O, 9.2 mM KBr, 9.4 mM NaCl, and 0.37 mM KI, pH 7.5). PAA converts HPB halides into halogens. These subsequently react with DPD to produce a compound that absorbs strongly at 515 nm. The increase in absorbance at 515 nm is proportional to the PAA content of the mixture (H_2_O_2_ does not produce any significant measurable absorbance in this assay). To assay for H_2_O_2_, the aliquots were combined with potassium iodide (10%) in the presence of sodium molybdate (3%), forming triiodide through oxidation by H_2_O_2_. The content of triiodide and, by extension, the original content of H_2_O_2_ were then determined through titration with thiosulfate (0.01 M), using starch as an indicator.

### Data analysis.

Data describing spore killing as a function of time were fitted to a Weibull microbial survival model by using GinaFit software optimized for the analysis of nonlinear microbial inactivation data (19). We then used the parameters from these models to interpolate the average time required to reach a predetermined reduction in spore viability, e.g., a 90% reduction for *D* values, as described previously (7).

## RESULTS

### Spore killing by PAA, H_2_O_2_, and PAA and H_2_O_2_ in combination.

**FIG 1:**
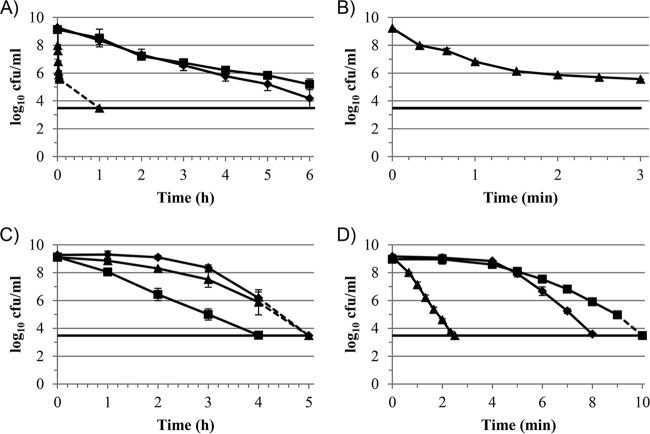
Killing of B. subtilis spores by treatment with different oxidizing agents. ◆, PS533 (WT); ■, PS578 (α^−^ β^−^); ▲, PS3394 (CotE^−^). Symbols represent mean values (for PS533, *n* = 5 except for in panel D, where *n* = 3 for the 5- and 7-min time points; for PS578, *n* = 4 except for in panel D, where *n* = 2 for the 5-, 7-, 9-, and 10-min time points; and for PS3394, *n* = 4 except for in panels A and B, where *n* = 2, and panel D, where *n* = 2 for the 0.66-, 2.33-, and 2.5-min time points), and error bars represent the standard deviations of the means. Dashed lines represent data points which fell below the limit of detection at the time indicated. Solid horizontal lines represent the limit of detection. (A) Treatment with 0.05% PAA. (B) Data for strain PS3394 shown in panel A, but with a modified *x* axis. (C) Treatment with 1% H_2_O_2_. (D) Treatment with P/H (0.05% PAA in combination with 1% H_2_O_2_).

Spores of all three strains of B. subtilis, PS533, PS3394, and PS578, were tested to assess their susceptibility to PAA (0.05%), H_2_O_2_ (1%), and P/H (a combination of 0.05% PAA and 1% H_2_O_2_) (Fig. 1). As observed previously (7), the WT and α^−^ β^−^ strains were the most resistant to PAA, whereas the CotE^−^ strain was extremely sensitive to this treatment (Fig. 1A). The resistance of each strain to PAA, ranked from most to least resistant, was PS578 > PS533 > PS3394 (Fig. 1A). The kill curve for CotE^−^ spores treated with PAA showed a distinct tail (Fig. 1B), as observed previously (7); however, after 60 min, there were no detectable survivors (Fig. 1A). WT and CotE^−^ spores were the most resistant to H_2_O_2_ treatment, with CotE^−^ spores being the slightly more susceptible of the two, and as expected, the α^−^ β^−^ spores, which lacked most of the DNA-protecting SASPs, were the most susceptible spores (Fig. 1C) (20).

The P/H combination was by far the most rapidly sporicidal of the three microbicides tested. WT spores treated with P/H dropped below the level of detection after only 8 min, compared with 5 and >6 h for H_2_O_2_ and PAA, respectively (Fig. 1). Of the three strains tested, PS578 was the most resistant to P/H, followed by PS533, with PS3394 being the most sensitive strain (Fig. 1D).

### Potentiation of PAA and H_2_O_2_.

In order to begin to better understand the interaction of the two biocides in combination, we compared the relative abilities of PAA to potentiate the activity of H_2_O_2_ and *vice versa* by using a checkerboard design and spores of strain PS533. The addition of lower (<0.04% [wt/vol]) concentrations of PAA had a relatively minor effect on the sporicidal activity of H_2_O_2_, as the concentration exponent (η) of H_2_O_2_ hardly changed relative to that for systems containing H_2_O_2_ alone (Table 1). Conversely, the addition of even the lowest concentration of H_2_O_2_ tested (0.1% [wt/vol]) to PAA considerably reduced the η value for PAA (Table 2), indicating that H_2_O_2_ had a far more profound effect on the activity of PAA than PAA did on the activity of H_2_O_2_.

**TABLE 1:**
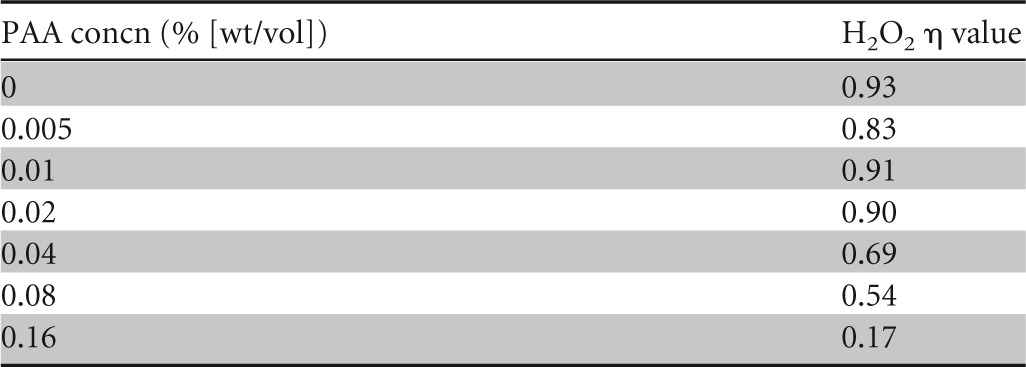
Concentration exponents (η) of H_2_O_2_ in the presence of various concentrations of PAA against spores of B. subtilis strain PS533

**TABLE 2:**
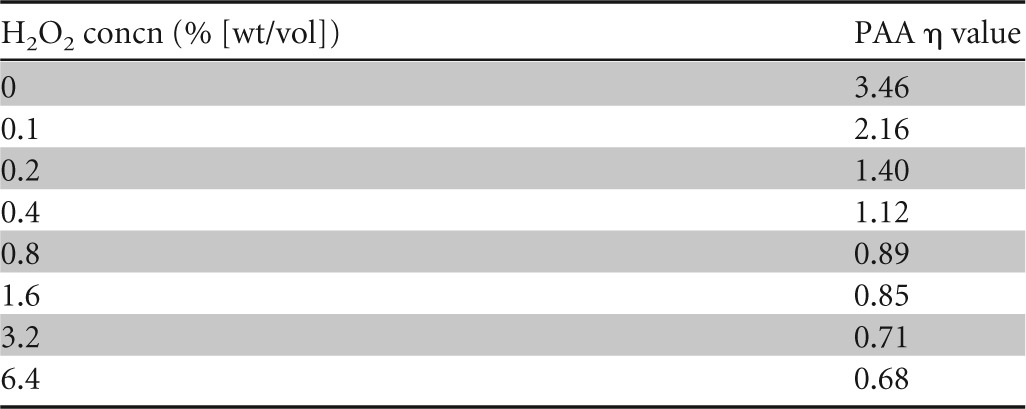
Concentration exponents (η) of PAA in the presence of various concentrations of H_2_O_2_ against spores of B. subtilis strain PS533

### Addressing the possibility of a shifting equilibrium of PAA and H_2_O_2_.

Compared to the WT spores, CotE^−^ spores were greatly sensitized to PAA and to P/H but not to H_2_O_2_, whereas α^−^ β^−^ spores were sensitized to H_2_O_2_ but not to either PAA or P/H (Fig. 1). Thus, the susceptibilities of the three strains to P/H match those to PAA more closely than those to H_2_O_2_, implying that the mechanism of activity is more similar to that of PAA than to that of H_2_O_2_. Given the similar susceptibility profiles of the three strains for PAA and P/H and the fact that H_2_O_2_ potentiated the activity of PAA more than PAA did that of H_2_O_2_, a possible explanation for the enhanced activity of the P/H combination is the generation of PAA from excess H_2_O_2_ and residual acetate, as suggested previously (8). Acetate is invariably included in solutions of PAA as a stabilizer. We therefore assayed both PAA and H_2_O_2_ at a range of concentrations (1%:0.05%, 3%:0.05%, and 1%:0.15% [H_2_O_2_:PAA]) during a 15-min exposure to WT spores. Despite the sporicidal activity of the various P/H combinations, the concentrations of both microbicides varied little over the 15-min test period (Table 3).

**TABLE 3:** Biocide concentrations measured during spore treatment with various combinations of H_2_O_2_ and PAA

Time (min)	Mean (SD) target biocide concn^*a*^					
	1% H_2_O_2_–0.05% PAA		1% H_2_O_2_–0.15% PAA		3% H_2_O_2_–0.05% PAA	
	H_2_O_2_	PAA	H_2_O_2_	PAA	H_2_O_2_	PAA
0	1.06 (0.04)	0.047 (0.00)	1.10 (0.04)	0.142 (0.005)	3.27 (0.02)	0.048 (0.00)
5	1.03 (0.02)	0.045 (0.001)	1.07 (ND)	0.137 (0.003)	3.14 (0.07)	0.046 (0.001)
15	0.99 (0.03)	0.043 (0.001)	1.07 (0.01)	0.139 (0.000)	3.18 (0.06)	0.045 (0.001)

a For the 0- and 15-min time points, *n* = 2; and for the 5-min time point, *n* = 4 for 1% H_2_O_2_–0.05% PAA, *n* = 1 for H_2_O_2_ and *n* = 2 for PAA in 1% H_2_O_2_–0.15% PAA, and *n* = 2 for 3% H_2_O_2_–0.05% PAA. ND, not determined.

### Susceptibility of pretreated spores to PAA or H_2_O_2_ treatment.

**FIG 2:**
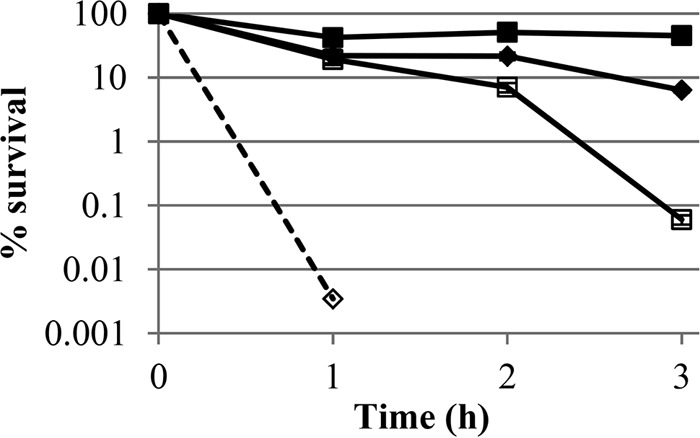
Sensitization of B. subtilis spores (strain PS533) to killing by PAA or H_2_O_2_. Symbols represent mean values (*n* = 3), and error bars represent the standard deviations of the means. Spores were either pretreated with PAA (0.05%; □) or H_2_O_2_ (1%; ♢), to give an approximately 90% (1 log) kill, or underwent no pretreatment (◆ and ■). Following pretreatment, microbicides were neutralized thoroughly, and the spores were treated with either PAA (0.05%; ◆ and ♢) or H_2_O_2_ (1%; ■ and □). The dashed line represents a data point which fell below the limit of detection at the time indicated. Spores used in this experiment were prepared independently from those used for Fig. 1 but are the same as those used for Table 4.

We investigated the relative importance of each individual microbicide to the activity of the two in combination by pretreating WT spores with either PAA (0.05%) or H_2_O_2_ (1%) to give an approximately 1-log_10_ (90%) reduction in viability. The microbicide was then thoroughly neutralized and the spores retested for susceptibility to H_2_O_2_ (for PAA-treated spores) or PAA (for H_2_O_2_-treated spores). Pretreatment with PAA slightly sensitized spores to a subsequent H_2_O_2_ treatment; however, pretreatment with H_2_O_2_ considerably sensitized spores to a subsequent PAA treatment (Fig. 2).

As H_2_O_2_ pretreatment severely compromised the spores' resistance to PAA, we investigated this effect further by pretreating WT spores with H_2_O_2_ (1%) for exposure times between 1 min and 2 h, none of which resulted in significant reductions in spore viability (results not shown), and then assessed their resistance to a subsequent PAA (0.05%) treatment. Spores were sensitized to PAA following very short exposures to H_2_O_2_, with pretreatments of 1 and 10 min reducing the *D* value for PAA (relative to that of an untreated control) 20 and 60%, respectively (Table 4). After 2 h of H_2_O_2_ pretreatment, the *D* value for PAA was reduced 89% relative to that of the untreated control (Table 4).

**TABLE 4:**
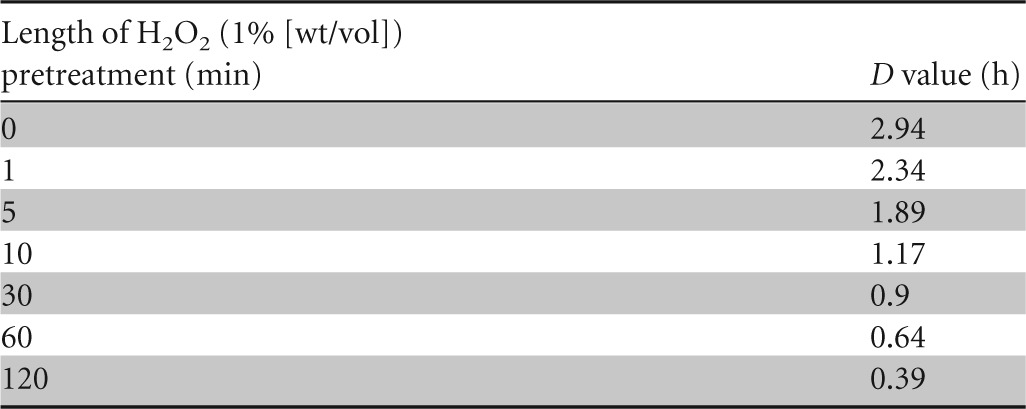
*D* values for spores of B. subtilis PS533 (with and without H_2_O_2_ pretreatment) treated with PAA (0.05%)^*a*^

a The spores used to generate these data were prepared independently from those used for Fig. 1 but are the same as those used for Fig. 2.

## DISCUSSION

In comparing the resistance profiles of WT, CotE^−^, and α^−^ β^−^ spores for the microbicides/microbicide combination used in this study, we observed greater similarities between PAA and P/H than between H_2_O_2_ and P/H; CotE^−^ spores were extremely sensitive to PAA and P/H but not to H_2_O_2_, whereas α^−^ β^−^ spores were highly susceptible to H_2_O_2_ but not to either PAA or P/H. This suggests that despite the presence of both microbicides in the P/H mixture at concentrations identical to those tested individually, it was PAA which exerted the greatest antimicrobial activity within the P/H combination. Why should PAA, not H_2_O_2_, be responsible for the majority of the activity of the P/H combination? It has been suggested that a likely reason for “synergy” between these two microbicides is the generation of PAA from H_2_O_2_ and residual acetic acid, which is invariably present in PAA solutions in order to improve its stability (8). However, neither the PAA nor H_2_O_2_ concentration varied greatly during spore treatments in this study, suggesting that little, if any, PAA was generated in the test system during spore treatment.

We therefore looked for a more subtle interaction between these two microbicides. Spores were greatly sensitized to the sporicidal activity of PAA following even short (<10 min), and entirely nonlethal, pretreatment with H_2_O_2_, while prolonged PAA pretreatment (>1 h) only slightly sensitized spores to H_2_O_2_. The observation that H_2_O_2_ exerted a nonlethal activity against bacterial spores which nevertheless significantly sensitized them to a subsequent PAA treatment suggested that H_2_O_2_ may have acted to facilitate the passage of PAA through the outer spore layers to its target site, which is thought to be the spore membrane (7). This would be analogous to the situation found for combinations of organomercurials and phenolics against vegetative organisms, where the phenol disrupts the cytoplasmic membrane, improving access of the mercury compound to the cell interior, where the compound exerts its lethal activity (21).

Treatment of spores with H_2_O_2_ and other oxidizing agents, such as hypochlorite, can remove proteins from the spore coat (22–24). It is therefore possible that in combination with PAA, H_2_O_2_ acts to strip proteins from the spore coat, effectively removing one of the principal barriers to PAA killing (7) and decreasing the time required for PAA to exert a sporicidal effect. However, we showed that spores became sensitized to PAA after only very brief exposures to a low concentration of H_2_O_2_. A concentration of 1% may not have been sufficient to remove significant amounts of coat protein (22). It has been suggested previously that the spore coat may provide resistance to oxidative microbicides by serving as a “reactive armor,” effectively reacting with and neutralizing the microbicide before it can penetrate further into the spore (25). CotA is a laccase which has been shown to play a role in spore H_2_O_2_ resistance (26). Other coat proteins, such as SOD and others (discussed in reference 27), could potentially detoxify oxidizing agents, such as PAA and H_2_O_2_, although none have been shown to protect specifically against these chemicals. We did not, however, gather evidence that detoxification was an important mechanism in the spores we studied. We therefore speculate that H_2_O_2_ rapidly interacts with reactive sites within the spore coat without necessarily removing significant amounts of protein, thus allowing PAA to pass through to react with other targets within the spore. Finnegan et al. described the reaction of H_2_O_2_ with different amino acids (28), which provides information as to what interactions could be expected within the spore coat. It would certainly be of interest to study the spore coat further to map out exposed amino acids.

Assuming that the synergistic activity of PAA and H_2_O_2_ is dependent upon a weakening of the spore coat defensive barrier, we may also expect hypochlorite to show a similar synergistic activity in combination with H_2_O_2_, since, like PAA, hypochlorite is most active against spores which lack a complete spore coat (29). Indeed, Marquis et al. (30) showed some synergy between hypochlorite and H_2_O_2_; however, they did not state the pH of the solutions tested, and because hypochlorite is considerably more active at lower pHs, one must consider that the effect of H_2_O_2_ lowering the pH of the system may have been responsible for the enhanced activity of these two biocides in combination.

The changes in concentration exponents (η) of the two active compounds in combination provide further support for our proposed mechanism of synergy. When the compounds were used alone, the η values of PAA and H_2_O_2_ were 3.46 and 0.93, respectively, indicating that these two biocides interacted with their respective targets quite differently, i.e., with H_2_O_2_ having a direct interaction and PAA having a weaker, less direct interaction with its target (31). However, in the presence of even the lowest concentrations of H_2_O_2_ tested, the η value for PAA dropped considerably, bringing it into the range of <2. This implies that in the presence of H_2_O_2_, PAA has a more direct interaction with its target site than when it is used alone. Leggett et al. (7) observed that WT and CotE^−^ spores also had differing η values for PAA (3.46 and 1.43, respectively), suggesting that in the absence of a complete spore coat, PAA had better, more direct access to its target site. It therefore seems likely that the synergistic interaction of PAA and H_2_O_2_ is a result of H_2_O_2_ enhancing the penetration of PAA through the spore coat, thus making PAA the principal sporicidal agent within the P/H mixture.

In this study, we found the interaction between PAA and H_2_O_2_ to be synergistic, with the sporicidal activity of the combination being largely due to PAA, not H_2_O_2_. The spore coat was the main spore resistance factor against this combination, and it is this barrier which is thought to be compromised by the activity of H_2_O_2_.
